# Effects of Low‐Load Blood‐Flow Restriction Training Versus High‐Load Resistance Training on Neuromuscular Performance and Neuromuscular Activation

**DOI:** 10.1111/sms.70203

**Published:** 2026-01-07

**Authors:** Romina Ledergerber, Paul Ritsche, Eric Lichtenstein, Luisa Prechtl, Oliver Faude, Martin Keller

**Affiliations:** ^1^ Department of Sport, Exercise and Health University of Basel Basel Switzerland

**Keywords:** blood flow restriction, middle‐aged adults, muscle strength, neuromuscular performance, rate of force development, resistance training, voluntary activation

## Abstract

Low‐load blood‐flow restriction (BFR) training is a potential alternative to high‐load (HL) resistance training, especially when mechanical stress must be minimized. However, its effects on neuromuscular activation remain unclear. This randomized controlled trial compared changes in voluntary activation (VA) and neuromuscular performance following 8 weeks of BFR versus HL knee extensor training and examined the effects of a subsequent 2‐week HL phase in both groups. Thirty‐seven healthy adults (37–59 years, 22 female) underwent progressive BFR or HL training for 8 weeks (phase 1), followed by 2 weeks of HL training on knee extensor muscles (phase 2). Outcomes included VA, maximal isometric and dynamic leg extension and leg press strength, rate of force development (RFD), and jump performance. Linear mixed models were used to analyze group*time interactions; Cohen's *d* effect sizes are reported. After both training phases, the BFR group showed smaller improvements than HL in VA (*d* = −0.31 to −0.37), maximal isometric strength (*d* = −0.07 to −0.27), dynamic strength (*d* = −0.18 to −0.75), and RFD (*d* = −0.48 to −0.54). Jump performance showed trivial between‐group differences (*d* = −0.01 to −0.05). Although a subsequent 2‐week HL phase improved outcomes in the BFR group, it did not fully restore neural adaptations to the level of continuous HL training. These findings underscore the essential role of mechanical loading in optimizing neuromuscular function. While BFR may serve as a useful preparatory method in contexts where high loads are initially contraindicated, follow‐up HL training is required to maximize neuromuscular adaptation.

**Trial Registration:** This study was preregistered on the Open Science Framework (DOI: 10.17605/OSF.IO/DA6SV)

## 1 Introduction

Regular resistance training of all major muscle groups has a positive effect on musculoskeletal health and is recommended at least twice weekly for the age category between 18 and 65 years [1]. For the majority of healthy adults, resistance training with moderate to high loads (70%–85% of the individual one repetition maximum, 1‐RM) is an appropriate means to increase both muscle mass and maximal strength [2]. It is nowadays well accepted that mechanical tension, but also metabolic stress, are highly relevant for gains in muscle mass and strength [3]. However, the high exposure to mechanical stress on joints is often contraindicated in many fields of rehabilitation [4]. To address patients with acute or chronic conditions of the musculoskeletal system, blood‐flow restriction (BFR) training has therefore gained attention in clinical research [4, 5]. BFR training is usually performed using low loads (LL) (20%–40% 1‐RM) while a cuff is applied at the proximal end of the trained limb [6]. The general idea of BFR training is to reduce the mechanical load but to increase the metabolic stress by occluding the venous return from the trained limb(s) [6]. A recent meta‐study showed the effectiveness of this training regimen with comparable adaptations for hypertrophy and similar or slightly lower adaptations in maximum strength when compared to high‐load resistance training [7].

While numerous intervention studies investigated the adaptations for muscle strength and muscle mass, neural adaptations—i.e. the ability to recruit muscle fibers simultaneously and with a high frequency—due to BFR training remain poorly understood. Investigating neural adaptations alongside muscular adaptations with training is, however, relevant, because increases in muscle strength are mainly explained by gains in muscle mass or neural adaptations or a combination of both [8]. Duchateau et al. [3] hypothesized that mechanical stress during resistance training is the driving factor for neural adaptations, while the metabolic stress likely has only a minor impact on neural adaptations. In line with this, Colomer‐Poveda et al. [9] showed that 4 weeks of LL training with or without BFR did not cause changes in neural drive and motoneuronal excitability, indicating that neural adaptations are not evident after LL‐BFR training. In contrast, a meta‐analysis summarized that neural adaptations obtained with electromyography following LL resistance training are facilitated with the use of simultaneous BFR [10]. In addition, the meta‐analysis revealed that enhanced levels of muscle excitation after LL‐BFR training were either similar or only slightly lower than after HL training. However, the authors also highlighted that studies using more sophisticated neurophysiological methods remain scarce, highlighting the need for further studies to investigate neural adaptations in response to long‐term training with BFR. The first aim of this study was therefore to assess neuromuscular performance and voluntary activation after either HL or LL‐BFR training. We hypothesized that HL training would result in enhanced performance and muscular activation when compared to LL‐BFR.

Additionally, we aimed to investigate whether a brief period of HL training following LL‐BFR further enhances muscular activation and, consequently, performance. This approach is clinically relevant because LL‐BFR can serve as an entry point when high loads are initially not feasible (e.g., due to joint or tendon limitations). After such a preparatory phase, patients may be better able to tolerate HL loading, which is known to be the key stimulus for neural adaptations. Even a few sessions of HL training have been shown to elicit meaningful neuromuscular changes [11]. Thus, we aimed to investigate whether a subsequent HL phase following LL‐BFR could restore neural adaptations to a level comparable to continuous HL training.

## 2 Methods

### 2.1 Experimental Approach to the Problem

This randomized controlled trial aimed to compare changes in neuromuscular activation following 8 weeks of HL versus LL‐BFR training (phase 1), as well as subsequent 2 weeks of HL training that was performed by both groups (phase 2). An identical test battery was therefore conducted four times: first, a familiarization before the baseline measurement (T0) was conducted, then changes were examined after 8 weeks of training phase 1 (T1) and after the 2 weeks of training phase 2 (T2), respectively. At least 48 h of recovery from the last training session was ensured before T1 and T2. Persons involved in data acquisition were blinded to group allocation. Coaches and study participants could not be blinded about group allocation, as BFR training with LL is easy to distinguish from HL training. According to the preregistration (DOI: 10.17605/OSF.IO/DA6SV), the outcomes included strength parameters for the knee extensors (maximum voluntary strength and rate of torque development), maximum leg press strength and rate of force development, vertical jump power, voluntary activation (VA), muscle cross‐sectional area of the rectus femoris, vastus lateralis and medialis muscle, architecture of the vastus lateralis muscle, patella tendon cross‐sectional area and stiffness, as well as anthropometry. However, for this manuscript, the muscle and tendon parameters were not considered and will be published separately. All measurements as well as the supervised training sessions were conducted at the Department of Sport, Exercise and Health of the University of Basel, Switzerland. The study was approved by the local ethics committee (Project‐ID: 2023‐01546) and all procedures were performed according to the Declaration of Helsinki and Good Clinical Practice.

### 2.2 Participants' Characteristics

A target sample of 40 healthy males and females (aged between 35 and 60 years) with no resistance training experience in the last 2 years was aimed for. Participants were ineligible for inclusion if they had hypertension (> 140/90 mmHg), were smokers, or had any acute or chronic condition that hindered lower‐body resistance training and testing. Further, they should not have any chronic cardiovascular disease, drepanocytosis, thrombosis or history of thrombosis, family history of epilepsy, hypertension, wearing electronic implants, intracranial metal particle, pre‐existing conditions of the central or peripheral nervous system, any acute or chronic injuries of the lower extremities, if they were pregnant, or consume Acetylsalicylic Acid (ASA) or other medication that promotes hemorrhaging. Before visiting the laboratory, they filled out a pre‐screening questionnaire asking for these criteria, while also securing that they had no resistance training experience in the last 2 years and would be able to follow the study procedures. At the laboratories, they provided written informed consent and underwent a medical screening including a resting electrocardiogram, blood pressure and consultation with a physician.

**FIGURE 1:**
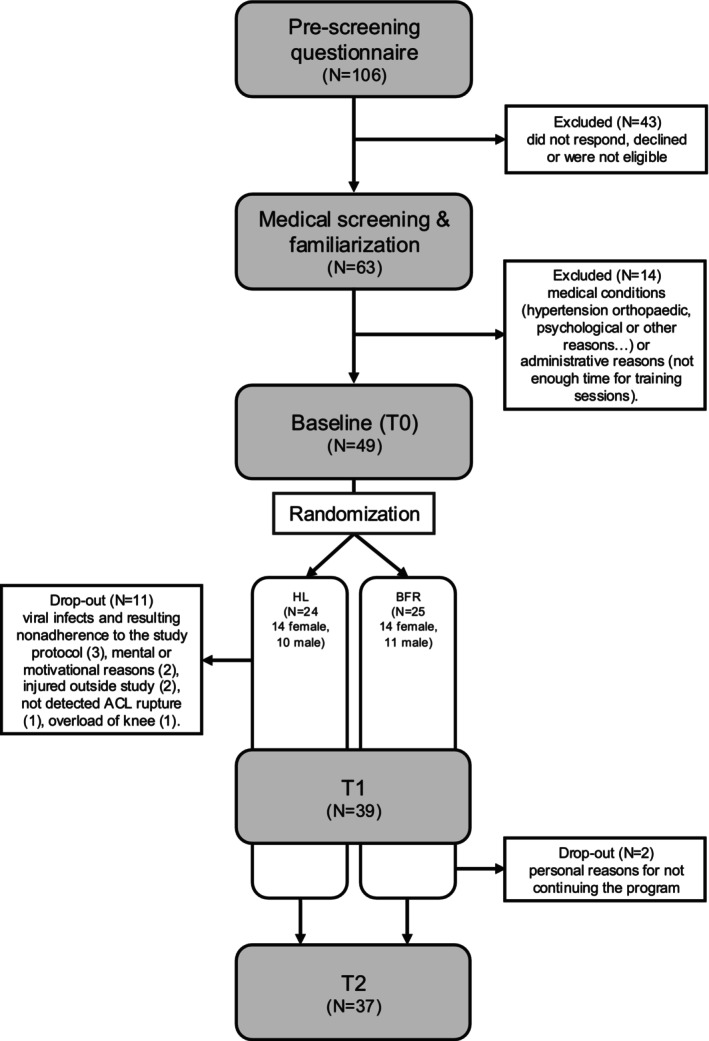
Flow chart of the study.

As shown in the flow chart in Figure 1, a total of 106 individuals registered and completed the online pre‐screening questionnaire through continuous enrolment. Of these, 63 attended the medical screening and familiarization session, after which 14 participants were excluded.

The remaining 49 participants were stratified into two intervention groups by a blinded statistician using the minimized means method, based on sex and body‐mass‐normalized leg press strength. Since the study aimed to compare two active training interventions, no additional resting control group was included. Reasons for dropout during the training phases are detailed in Figure 1. The final sample included 37 healthy, middle‐aged adults for complete analysis (see Table 1 for participant characteristics).

**TABLE 1:** Baseline characteristics.

	BFR		HL	
	M (*N* = 11)	F (*N* = 12)	M (*N* = 5)	F (*N* = 10)
Age [y]	47.3 ± 6.0	49.7 ± 7.2	45.0 ± 6.1	47.3 ± 5.9
Body mass [kg]	84.9 ± 13.7	72.2 ± 10.7	81.3 ± 11.4	73.1 ± 15.2
BMI [kg/m^2^]	25.3 ± 4.0	27.1 ± 3.9	28.0 ± 4.0	27.0 ± 6.5
Skeletal muscle mass [kg]	37.7 ± 4.2	25.9 ± 2.7	35.9 ± 2.4	27.2 ± 2.1
Body fat [kg]	18.3 ± 7.8	24.9 ± 9.8	17.8 ± 8.4	23.8 ± 13.1
Leg press strength [N]	4115.9 ± 1095.9	2482.0 ± 531.3	4459.2 ± 921.5	2544.2 ± 647.8
Leg press strength [N/kg]	49.8 ± 12.1	35.1 ± 10.2	53.7 ± 12.1	35.6 ± 12.6

Abbreviation: BMI, body mass index.

### 2.3 Experimental Procedures

#### 2.3.1 Neuromuscular Performance

Measurements of neuromuscular performance, defined as the mechanical output resulting from the integrated neural and muscular contributions to force generation, included the isometric knee extension strength, isometric leg press strength, and rate of force development, and a countermovement jump.

The maximal isometric knee extension strength was measured using a dynamometer (ScienceToPractice LtD, Ljubljana, Slovenia). The participants were fixed with straps in an upright position (90° hip flexion) and a knee angle under tension of 60°. Maximal leg press strength and rate of force development (RFD) were measured using an isokinetic dynamometer (D&R Ferstl, IsoMed 2000, Hemau, Germany). The participants were fixed with a back inclination of 45° and a knee angle under contraction of 110°. Both measurements were performed according to international guidelines. Participants were instructed to extend their leg as fast and as forcefully as possible against the resistance and to maintain a plateau for approximately 3 s [12]. The maximal isometric strength of leg extension and leg press was determined by the peak force achieved in each trial. The initial increase in force within 150 ms of the leg press was considered a reliable measurement interval for RFD [12]. RFD was included due to its sensitivity to neural adaptations and its relevance for rapid force production in functional tasks [12].

The countermovement jump (CMJ) was performed on a force plate (Leonardo Mechanograph, Novotec Medical GmbH, Germany) in order to examine the potential transfer of machine‐based strength training to complex multi‐joint movements. The participants were asked to jump as high as possible from a dynamic squat with arms akimbo. Maximal jump height was estimated from the impulse applied during the jump. Maximal power was obtained from the highest acceleration, normalized to body mass.

All measurements were repeated three times with a rest of 1 min in between. The peak force, the highest RFD, the maximum power, and jump height of each measurement respectively were taken into account in the analysis.

#### 2.3.2 Voluntary Activation

Voluntary activation (VA) of the leg extensor muscles was measured using the Interpolated Twitch Technique. Respecting the recommendations from the recent Delphi survey with expert consensus [13], we performed electrical muscle stimulation as a valid method to determine VA. Two self‐adhesive electrodes (7.5 × 13 cm, oval, PALS, Axelgaard Manufacturing co. Ltd., Denmark) were attached proximally and distally to the quadriceps femoris muscle. A constant current electrical stimulator (Digitimer DS7R, Hertfordshire, UK) was used to deliver single and paired electrical square‐wave pulses (pulse width 1 ms). The individual optimal stimulation intensity was determined by increasing the stimulation intensity until maximal twitch responses were obtained. The optimal stimulation intensity was considered to be reached when an increase of 3 consecutive 50 mA increments did not lead to any increase in the evoked twitch force (273 ± 103 mA). To guarantee suprathreshold stimulations during data acquisition, all subsequent stimuli were applied at 120% of the individual plateau (343 ± 102 mA) using paired pulses (1 ms, 100 Hz).

In the following, 3–5 MVCs were performed using the identical setup as described for maximal isometric knee extension strength. Superimposed doublet twitches were applied while participants performed MVC with visual online feedback about the applied torque shown on a screen. Superimposed twitches were applied manually during each MVC, while a resting doublet was delivered unexpectedly 2–4 s after the first contraction. To account for underperformance at the point of stimulation, the superimposed twitch was corrected according to the percentage force deficit compared to the MVC [14]. Using the corrected superimposed twitch of the highest force plateau, this further allowed the quantification of VA, by the formula VA (%) = (1 − (superimposed twitch/resting twitch)) × 100 [15]. The perception of discomfort was rated using the visual analogue scale. The rating from 0 to 10 was used, where 0 indicated a very low, 10 a very high level of pain, resp.

#### 2.3.3 Anthropometry

Body height was measured to the nearest 0.1 cm using a stadiometer. A bioimpedance analysis was conducted using the Inbody 720 (Biospace Co. Ltd., Seoul, Korea) to quantify body composition.

#### 2.3.4 Training Intervention

For 10 weeks, the participants took part in a total of 20 supervised progressive resistance training sessions with at least 1 day rest between consecutive sessions. On average, they trained twice a week for 45–60 min per session. In training phase 1 (weeks 1–8), they performed either LL‐BFR training or HL resistance training, while in phase 2 (weeks 9 and 10) both groups participated in the identical HL training.

In each training session, they were asked about their health status or whether any symptoms had developed after the training sessions to monitor possible adverse events. The training sessions began with a warm‐up on a cycle ergometer with a load corresponding to the body weight (1 W/kg). Every 2 weeks and in the last session, the 1‐RM was tested to progressively adjust the training load. The 3 exercises, leg press, leg curl, and leg extension, were always performed in the same order and with a full range of motion. Four sets of each exercise were performed with a rest of 1 min between sets and 3 min between exercises.

The high‐load training in phase 1 was carried out according to the international ACSM guidelines [1]. Starting from 70% 1‐RM the training load was gradually increased by 5% of the absolute 1‐RM, which was re‐assessed every 2 weeks. Repetitions were adjusted accordingly, resulting in 2‐week training blocks of 70% 1‐RM and 12 repetitions, 75% and 10 repetitions, 80% and 8 repetitions, and 85% and 6 repetitions, respectively.

The BFR training sessions were performed in accordance with the recommendations for safe and efficient use of BFR [6]. Comparable to HL training, the training load in the BFR group was also increased by 5% every 2 weeks but starting from 20% 1‐RM and ending with 35% 1‐RM. The first set was always performed with 30 repetitions; in sets 2–4, only 15 repetitions were performed. During each training session with BFR, a 12‐cm‐wide cuff connected to a pneumatic tourniquet system (Tourniquet Touch TT20; VBM Medizintechnik GmbH, Sulz am Neckar, Germany) was worn bilaterally at the most proximal portion of each thigh. Before each training session, the arterial occlusion pressure (AOP) was determined individually while participants maintained a sitting position. To determine AOP, the cuff pressure was increased stepwise in 5 mmHg increments until the arterial pulse at the posterior tibial artery was no longer detectable by Doppler ultrasound (Handydop; Kranzbühler, Solingen, Germany). The cuff pressure needed for arterial occlusion was defined as 100% of AOP. During exercise, the cuff pressure was set to 50% of AOP. The cuff was kept inflated during the rest periods between sets but was released between exercises.

In the second training phase (weeks 9 and 10), all participants completed HL training with 85% of 1‐RM and 6–8 repetitions.

If less than 80% of the desired repetitions could be performed in a set, the load was adjusted to better achieve the number of repetitions. Perceived exertion after each exercise was assessed using the modified Borg scale [1, 2, 3, 4, 5, 6, 7, 8, 9, 10]. To increase compliance, all participants were offered free use of the gym for upper body exercises.

### 2.4 Data Analysis and Statistical Analysis

Data collection was performed with IMAGO Record (version 8.64, pfitec, Endingen Germany). Descriptive statistics were used to present mean and standard deviation (mean ± SD) as well as percent changes between T0–T1 and T1–T2 of all variables, but only for complete cases. Linear mixed models with random effects were used to assess the interaction effect of *group* (HL, BFR) and *time* (T0, T1, T2) on all outcomes, with *sex* and age included as covariates. *p*‐values were calculated for the linear mixed models, with statistical significance defined as *p* < 0.05. The magnitude of changes is reported with effect sizes while the 95% Confidence Intervals provide a good estimator for the precision of the effect [16]. Effects below 0.2 are considered trivial, between 0.2–0.5 small, 0.5–0.8 moderate, and > 0.8 as large. The percentage changes from the model output were calculated as percentage changes from T0 (set at 100%). All statistical analyses and figures were performed with RStudio (version 4.2.0) using the package lme4 [17]. An anonymized version of the full dataset used for all analyses is openly accessible on the Open Science Framework: https://doi.org/10.17605/OSF.IO/FZ38R.

Although the primary aim of the familiarization session was to help participants get accustomed to the testing procedures, we also performed reliability analyses with the baseline (T0) data to estimate measurement agreement in our own setup. Inter‐session reliability was therefore described with Intra‐class Correlation Coefficient (ICCs), Coefficient of Variation (CoV) in % and Typical Error (TE) on the original scale with 90% CIs using a published spreadsheet [18]. The ICC values less than 0.50 indicate poor reliability, while values between 0.50 and 0.75 are considered moderate, 0.75–0.90 as good, and values above 0.90 indicate excellent reliability [19]. Furthermore, a CoV between 15% and 10% is considered moderate, < 10% as acceptable, < 5% as good reliability, respectively.

## 3 Results

Baseline characteristics of participants by training group (BFR vs. HL) and sex are shown in Table 1. The two groups were well balanced in terms of age, body composition, and body‐mass normalized leg press strength.

All participants completed the full 20 training sessions over the 10‐week intervention period. Adherence to the planned training protocol was high in both groups. On average, participants completed 98% in the BFR group (range 82%–106%) and 97% of the prescribed repetitions in the HL group (range 88%–100%), indicating good compliance with the intended training volume. The rate of perceived exertion averaged 6.4 on a 0–10 scale in BFR (range 5.2–8.7) and 7.8 in HL (range 7.5–8.4).

### 3.1 Neuromuscular Performance

**FIGURE 2:**
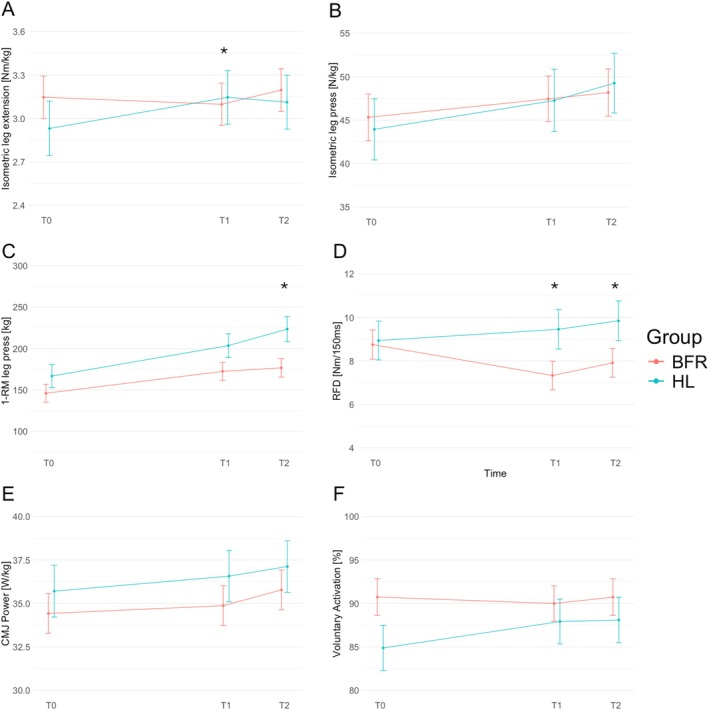
Key results visualized as estimated marginal means, separated for groups (BFR: Red lines; HL: Blue lines) and across time (T0, baseline; T1 after training phase 1; T2 after training phase 2): (A) body‐mass normalized isometric leg extension strength; (B) body‐mass normalized isometric leg press strength; (C) absolute 1‐repetition maximum of leg press strength; (D) Rate of force development (RFD) within first 150 ms of leg press strength; (E) body‐mass normalized maximal power of countermovement‐jump (CMJ); (F) Voluntary activation (VA). Significant interactions are marked with a * (*p* < 0.05).

**FIGURE 3:**
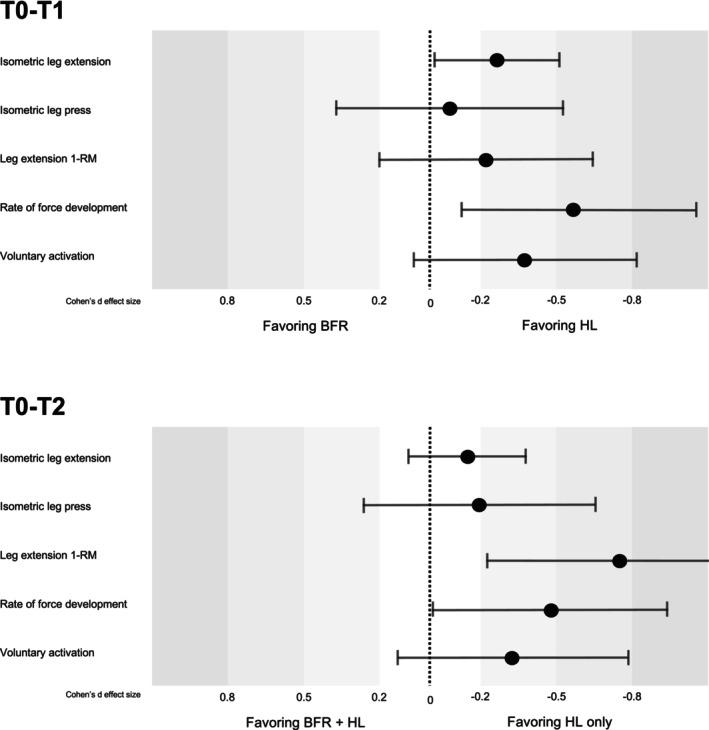
Modeled group*time interaction effects (Cohen's *d* ± 95% confidence intervals) for key neuromuscular outcomes between low‐load blood flow restriction (BFR) and high‐load (HL) resistance training. Effects are shown for changes from baseline to post‐phase 1 (T0–T1) and from baseline to post‐phase 2 (T0–T2). Positive values indicate greater improvements in the HL group compared to BFR.

Descriptive changes in neuromuscular performance across time for each group are presented in Table 2. Percent changes of these maximal and explosive performance outcomes are represented as Supporting Information S1 but only for complete cases. Figure 2 illustrates the estimated marginal means across time for each outcome, whereas Figure 3 summarizes the corresponding modeled group*time interaction effects (T0–T1 and T0–T2) with 95% confidence intervals.

**TABLE 2:** Descriptive statistics of all outcomes across time for each group.

	Group	BFR						HL					
	Outcome	N	T0, mean (SD)	N	T1, mean (SD)	N	T2, mean (SD)	N	T0, mean (SD)	N	T1, mean (SD)	N	T2, mean (SD)
Maximal isometric strength	Isometric leg extension [Nm]	21	240 (81)	21	239 (83)	20	251 (75)	15	213 (73)	15	229 (67)	15	227 (76)
	Isometric leg extension [Nm/kg]	21	3.2 (0.8)	21	3.1 (0.8)	20	3.2 (0.8)	15	2.9 (0.9)	15	3.0 (0.8)	15	3.0 (0.8)
	Isometric leg press [N]	20	3396 (1187)	21	3538 (1236)	19	3677 (1336)	14	3308 (1215)	12	3628 (1005)	14	3759 (1643)
	Isometric leg press [N/kg]	20	43 (13)	21	46 (14)	19	47 (13)	14	44 (14)	12	49 (13)	14	49 (19)
Maximal dynamic strength	Leg press 1‐RM [kg]	23	144 (56)	23	171 (77)	17	176 (70)	15	149 (64)	14	172 (62)	9	213 (105)
	Leg extension 1‐RM [kg]	23	84 (27)	21	102 (35)	14	107 (32)	15	72 (26)	14	97 (24)	7	111 (37)
	Leg flexion 1‐RM [kg]	23	56 (21)	19	66 (17)	16	67 (16)	15	47 (15)	13	61 (13)	9	72 (26)
Maximal explosive strength	Rate of force development [Nm·150 ms^−1^]	19	9.0 (3.2)	20	7.6 (3.6)	19	8.3 (3.9)	12	7.3 (3.3)	11	8.0 (3.0)	11	8.5 (4.1)
	Countermovement‐jump power [W/kg]	21	35 (6)	21	35 (6)	21	36 (6)	12	36 (10)	12	34 (7)	11	35 (7)
	Countermovement‐jump height [cm]	21	34 (8)	21	35 (8)	20	36 (10)	12	33 (11)	12	31 (8)	11	33 (7)
VA	Voluntary activation [%]	16	90.3 (6.8)	17	88.6 (10.7)	15	90.1 (7.7)	11	86.7 (9.7)	12	89.5 (7.9)	12	89.6 (7.2)

*Note:* Number of valid measures (N); mean (SD = standard deviation).

#### 3.1.1 Isometric Strength

Strength gains in the BFR group were small to negligible across all isometric outcomes with −1.6% to +5.9% change (see Supporting Information S1). In the HL group, small improvements were observed (+4.9% to 13.9%), particularly for isometric leg extension (+13.9%). A statistically significant negative interaction was detected for BFR at T1 (−14.4%; see Table 3, Figure 3), indicating that strength in the BFR group increased to a lesser extent compared to HL over this period. Other isometric strength measures showed non‐significant but consistent trends favoring HL over BFR at both T1 and T2. The estimated marginal means of body‐mass normalized isometric leg press and extension strength are visualized in Figure 2A,B.

**TABLE 3:** Model outcomes.

	Model	Model terms and effects for group*time Interaction in favor of BFR							
		T0–T1*group				T0–T2*group			
	Outcome	Estimate (SE)	Cohen's *d* (95% CI)	%change (95% CI)	*p*	Estimate (SE)	Cohen's *d* (95% CI)	%change (95% CI)	*p*
Maximal isometric strength	Isometric leg extension [Nm]	−21 (9)	**−0.27** (−0.51; −0.03)	−14.4 (−24.1; −4.7)	0.032	−13 (10)	−0.16 (−0.41, 0.08)	−9 (−18.8; 0.8)	0.191
	Isometric leg press [N]	−81 (274)	−0.07 (−0.52; 0.38)	−2.7 (−14.5; 9.1)	0.769	−223 (274)	−0.19 (−0.64; 0.26)	−5.2 (−17.2; 6.8)	0.419
Maximal dynamic strength	Leg press 1‐RM	−10 (10)	−0.18 (−0.51; 0.15)	−9.8 (−21.2; 1.7)	0.30	−26 (12)	**−0.44** (−0.05; −0.83)	−12.4 (−25.4; 0.6)	0.03
	Leg extension 1‐RM	−6 (6)	**−0.21** (−0.63; 0.2)	−21.0 (−34.8; −7.2)	0.32	−21 (8)	**−0.75** (−0.21; −1.3)	−35.2 (−52.8; −17.6)	0.01
Maximal explosive strength	Rate of force development [Nm·150 ms^−1^]	−1.9 (0.8)	**−0.54** (−0.99; −0.09)	−31.7 (−53.9; −9.6)	0.023	−1.7 (0.8)	**−0.48** (−0.03; −0.94)	−25.5 (−47.8; −3.1)	0.042
	Countermovement‐jump power [W/kg]	−0.4 (0.7)	−0.05 (−0.24; 0.13)	−0.8 (−4.7; 3.1)	0.576	−0.5 (4.2)	−0.01 (−0.20; 0.18)	−1.9 (−5.8; 2)	0.933
VA	Voluntary activation [%]	−3.4 (2.2)	**−0.37** (−0.82; 0.09)	−4.2 (−9.1; 0.6)	0.121	−2.9 (2.2)	**−0.31** (−0.79; 0.16)	−3.2 (−8.1; 1.8)	0.199

*Note:* Linear mixed‐effects model results for group*time interactions (univariate models with random slope and intercept for participants, adjusted for sex and age). Estimates represent between‐group differences in change over time; negative values indicate effects in favor of HL. Cohen's *d* (95% CI) quantifies effect magnitude. Values printed bold show at least small effects (*d* > 0.2).

#### 3.1.2 Dynamic Strength

Both groups improved in 1‐RM measures over time, but gains were generally greater in the HL group (+15.8% to 31.3%) than BFR (+16.2% to 20.5%), as visualized in Figure 2C. At T1, small effects of lower improvements in BFR compared to HL were noted (−9.8% to −21.0%), alongside T2, where significant moderate and large group*time interaction effects were found for both leg press and leg extension 1‐RM (−12.4% to −35.2%; see Table 3; Figure 3).

#### 3.1.3 Rate of Force Development

In the BFR group, RFD decreased from T0 to T1 (−15.0%) but partially recovered until T2 (T1–T2: +22%), as visualized in Figure 2D. In contrast, the HL group showed continuous improvement in RFD throughout the intervention. Significant moderate interaction effects favoring HL were observed at both T1 (−31.7%) and T2 for RFD (−25.5%, see Table 3 and Figure 3). A tendency for improvements but no meaningful differences between groups were observed for CMJ power or height, as visualized in Figure 2E and Table 3.

### 3.2 Neuromuscular Activation

VA decreased slightly in the BFR group at T1 (−1.1%) and showed a minimal increase following the subsequent HL training phase (T1 to T2: +1.8%). In contrast, the HL group demonstrated the greatest improvement in VA between T0 and T1 (2.9%) (see Figure 2F). A small effect in favor of HL training was observed at T1 (−4.2%), indicating superior neuromuscular adaptation compared to BFR. However, the two‐week HL training phase following BFR was insufficient to fully compensate for the attenuated neural adaptations, as shown by the persistent small effect favoring HL at T2 (−3.2%, see Table 3 and Figure 3). Perceived pain in response to electrical stimulations was on average 4.1 (2.7) and ranged from 0 to 8.9 on the scale from 0 to 10.

### 3.3 Reliability of Measurements

To estimate test–retest reliability between the familiarization and baseline (T0) measurements, we analyzed the agreement between both timepoints. Isometric leg extension strength showed excellent reliability (ICC = 0.93), with a moderate CoV of 14.2% and a typical error (TE) of 22.0 Nm [90% CI: 18.4; 27.7]. Isometric leg press strength demonstrated good reliability (ICC = 0.83), with an acceptable CoV of 8.2% and a TE of 222 N [90% CI: 184; 282]. Voluntary activation also showed good reliability (ICC = 0.85), with a good CoV of 4.1% and a TE of 3.5% [90% CI: 2.8; 4.8].

## 4 Discussion

This study investigated the effects of 8 weeks LL‐BFR compared to HL resistance training on neuromuscular performance and activation, followed by an additional 2 weeks of HL training in both groups. After training phase 1, HL training led to significantly greater improvements in neuromuscular performance and a small but consistent advantage in voluntary activation over BFR. Notably, a subsequent 2‐week period of HL training was insufficient to fully offset the attenuated neural adaptations observed in the BFR group. Together, the results suggest that the high mechanical loading in this study provided the critical stimulus for optimizing neuromuscular activation.

### 4.1 Neuromuscular Performance

#### 4.1.1 Maximal Strength

*Training phase 1*: Both HL and BFR training resulted in clear increases in maximal dynamic and isometric strength, with greater gains observed in the HL group after training phase 1. These results align well with the expected ranges of improvement and are consistent with results from previous studies [5, 20, 21]. The present 1‐RM improvements in both BFR (+16.2%–20.5%) and HL groups (+15.8%–31.3%) after 8 weeks are comparable to previous findings, where 34%–41% gains after HL and 13%–32% gains after LL‐BFR training in leg extension 1‐RM were reported over 6–10 weeks [20, 21]. Similarly, our moderate isometric strength gains fall in previously reported ranges, with HL again producing greater improvements than BFR [7, 9, 21]. Isometric strength improvements are less consistently reported in the literature, likely due to task dependency after dynamic strength training and the limited transferability between training and testing modalities [22]. Thus, the greater increases observed in dynamic compared to isometric strength are well in line with the literature given the principle of training specificity and adaptations that are specific to movement velocity and contraction mode [22]. Or—in other words—training characteristics during HL training (e.g., moving high loads) were similar to characteristics needed for 1‐RM assessments, which is likely the explanation for the more pronounced increases in dynamic strength after HL when compared to BFR training.

*Training phase 2*: The application of a sequential training program may be particularly promising, as it enables the combination of distinct training modalities tailored to individual capacity and progression. In this study, both groups showed further improvements in maximal strength after the 2‐week HL training phase. While dynamic strength improvements were more pronounced in the HL group, isometric strength gains at T2 were comparable between groups. One study has shown that relevant strength increases can be observed even with 3 HL training sessions (6.3% ± 4.5%) in wrist flexors [23]. Our findings are consistent with this, although the magnitude was slightly lower.

#### 4.1.2 Explosive Strength

*Training phase 1*: Explosive force as measured by RFD improved markedly with HL training but declined moderately in the BFR group during phase 1, resulting in a significant interaction. In contrast, gains in jumping power were similar between groups.

This aligns with prior work showing that HL and explosive resistance training are potent stimuli for RFD gains, likely due to increased neural drive and improved recruitment of fast motor units [24, 25, 26]. In line with this, the group*time interaction favoring HL at T1 supports the notion that high‐intent contractions under mechanical load are effective in enhancing rapid‐force production [24].

Interestingly, previous work by Nielsen et al. [27] reported that intense LL‐BFR training can temporarily impair contractile RFD, with reductions observed 5 days post‐intervention and recovery and improvement, respectively, only seen after 12 days. This transient decline was explained by delayed restoration of excitation‐contraction coupling and contractile function rather than persistent metabolic stress [27]. Since our T1 testing occurred within a similar window (> 48 h days post‐training), it is possible that RFD in the BFR group was acutely suppressed due to residual fatigue or reduced contractility, rather than a lack of adaptation per se. Therefore, our data cannot definitively distinguish whether the reduced RFD in the BFR group reflects attenuated neural adaptations, transient contractile impairment, or both.

In contrast to isometric RFD testings, the minimal change in CMJ performance without group differences likely reflects task specificity. While this multi‐joint movement is complex, a recent study showed additional improvements of CMJ performance when BFR was applied during complex training (primary plyometric exercises). As CMJ was not explicitly trained in this study, we assume that most improvements are likely to be attributed to familiarization rather than to actual transfer from machine‐based exercises [8, 28].

*Training phase 2*: Following the 2‐week HL phase, the BFR group showed a marked improvement in RFD. This may reflect a robust response to the mechanical loading stimulus, a recovery from previously reduced contractile function induced by the BFR training phase, or a combination of both. These findings are consistent with prior work indicating that RFD adapts rapidly to HL protocols, even after brief exposures [24]. Contrary to this, CMJ performance remained unchanged, likely due to the absence of a specific training stimulus for the motor demands of the multi‐joint task [26, 28].

### 4.2 Neuromuscular Activation

*Training phase 1*: It is well established that the initial phase of resistance training elicits large improvements in strength, primarily driven by neural adaptations rather than hypertrophy [29]. Although participants were untrained, only the HL group demonstrated increased VA after phase 1, whereas VA remained unchanged in the BFR group, resulting in a small interaction effect. In line with these findings, after 6 weeks of leg extension training Cook et al. [21] reported a small effect for an increase in central activation ratio following HL training (+3%) and a small decrease after LL‐BFR (−2%), although the sample size was very small and results were non‐significant (*N* = 3 per group). Our results therefore support the view that high mechanical loading is key to drive neuromuscular activation [3, 10]. However, it must be acknowledged that the literature on BFR‐induced neuromuscular adaptations remains sparse and inconsistent.

Mechanical load is considered the primary stimulus to increase recruitment efficiency and firing frequency of motor units—processes directly reflected in VA [22]. However, metabolic stress, while also a potent stimulus for strength gains, influences neuromuscular activation in a more indirect way. Recent work suggests that the accumulation of inorganic phosphates rather than acidosis is the dominant peripheral factor contributing to muscle fatigue [30]. Elevated Pi and related metabolic disturbances can activate group III/IV afferents, which in turn inhibit α‐motoneurons and modulate central motor output by reducing spinal excitability and altering cortical drive [30, 31]. Therefore, in theory at least, an unfamiliar stimulus arising from fatigue induced by low‐load BFR training could potentially trigger neuromuscular adaptations by sufficiently challenging existing motor unit recruitment patterns [32].

Indeed, acute studies comparing LL resistance exercise with and without BFR have shown that BFR increases the activation of motor units with higher action potential amplitude and may modulate corticomotor excitability through altered sensory feedback [33]. Thus, the implementation of BFR during exercise provokes markedly greater acute neuromuscular fatigue—encompassing central and peripheral components—than exercise conducted under unrestricted perfusion, thereby obliging the nervous system to recalibrate its efferent neural output, a compensatory process that may, in principle, precipitate long‐term adaptive responses [34]. However, chronic studies have reported mixed results. While some have observed increases in EMG amplitude or altered frequency bands following BFR training [20, 35, 36, 37], others have found no clear effects [9, 21, 32, 38]. Interestingly, when directly comparing LL‐BFR to HL resistance training, Kubo et al. [38] observed increased EMG activation only in the HL group after 12 weeks but no changes in the LL‐BFR group. Similarly, Biazon et al. [20] showed that EMG activity was higher following HL training and HL combined with BFR than with LL‐BFR, suggesting that mechanical load is the primary driver of neuromuscular activation. The lack of an additional effect of BFR under HL conditions supports the view that metabolic stress plays a secondary role when mechanical tension is already sufficient.

*Training phase 2*: As described further above, LL‐BFR training did not increase VA. Thus, the aim of the second training phase was to apply HL subsequently to LL‐BFR training in order to introduce a novel HL training stimulus in the BFR group. This sequential approach—initiating with LL‐BFR training to enhance muscle strength and mass under reduced mechanical stress, followed by HL training aimed at optimizing neuromuscular activation—was designed to capitalize on distinct neuromuscular adaptation mechanisms. However, our data suggest that 2 weeks of HL training was insufficient to result in comparable improvements in VA compared to the group that solely trained with HL.

Our findings align with prior evidence showing limited changes in VA following short‐term resistance training. Lee et al. [39] and Tillin et al. [40] both reported no meaningful improvements in VA after 4 weeks, despite signs of neural adaptation such as increased EMG and M‐wave amplitudes along strength improvements. They suggested that early strength gains may reflect enhanced recruitment or corticospinal transmission rather than increased voluntary drive [39, 40]—or in other words, VA measurements might not be enough sensitive for distinct neural alterations. Similarly, Mason et al. [23] observed transient increases in corticospinal excitability over 2 weeks of HL training, which did not accumulate across sessions, indicating that short‐term neural responses may not directly translate into lasting changes in VA. This is in line with a review concluding that early neural adaptations are largely due to improved corticospinal efficiency rather than increased maximal motor output [41].

Finally, we must acknowledge that the improvement ranges are small and within the expected data variations. Even if we cannot interpret the reliability results without emphasizing that a familiarization session is usually required for clean measurements, we assume that the true reliability of our devices is higher.

### 4.3 Methodological Considerations

The inclusion of a familiarization session strengthened the study quality and allowed for the estimation of measurement reliability. In line with previous reliability results, our reliability data were mostly within a good to excellent range; some degree of learning effect was expected, which may have biased the true reliability estimates.

For example, we found that, despite the ICC for isometric leg extension being excellent, the CoV was > 10%, which is beyond the accurate range. For the isometric leg press, the CoV is considered accurate, but the ICC is only considered ‘good’. Even if the reliability measurements included the familiarization session, we speculate that isometric measurements might not be the perfect indicator with which to interpret the effects on muscle strength.

Although our results show good reliability for VA, the Interpolated Twitch Technique presents specific methodological challenges [42]. In some cases, participants may have underperformed due to discomfort or anticipation of stimulation, leading to submaximal effort. To account for this, we applied a correction formula to approximate true VA values, although perfect accuracy cannot be assumed [14]. Moreover, a few participants refused VA measurements altogether, reducing the available sample size.

At this point, it is also important to note that the BFR group started with a higher baseline VA than the HL group, as we did not stratify the groups for this outcome. The higher initial VA in the BFR group potentially limited the capacity for further adaptation. Additionally, as the study involved middle‐aged adults, age‐related declines in neuromuscular function and performance were not yet pronounced [43]. In older adults, the potential for VA and strength improvements through both BFR or HL training might have been greater due to more pronounced baseline deficits.

The drop‐out rate of 22% was within the anticipated range; however, a disproportionate number of males from the HL group withdrew, leading to a slight imbalance in final group allocations. As sex‐related differences in strength adaptations cannot be fully excluded [44], this imbalance may have influenced the observed outcomes and should be acknowledged as a potential confounding factor. Additionally, participant age varied widely within the middle‐aged category. We also did not formally assess physical activity levels beyond excluding participants who had engaged in resistance training within the past 2 years. As a result, some participants were recreational endurance athletes, while others were relatively sedentary, potentially introducing variability that may have influenced the neuromuscular adaptations observed.

As the aim of this study was to compare two distinct training regimes, the protocols inherently differed in relative load and fatigue accumulation, resulting in different levels of perceived effort. These differences reflect the practical characteristics of HL and LL‐BFR training and should be considered when interpreting the outcomes. However, future studies could consider matching the volumes and relative intensities of the protocols more closely.

Finally, 1‐RM testing was performed every 2 weeks to adjust training loads individually. Although necessary for precise load progression, repeated maximal testing may have acted as a training stimulus. As repeated 1‐RM testing has been shown to elicit specific neuromuscular responses to this task, particularly for the BFR group, this could have influenced the results of the 1‐RM testing [45].

### 4.4 Perspective

This study extends current evidence on low‐load blood‐flow restriction (LL‐BFR) training by directly comparing it to high‐load (HL) resistance training with a focus on neuromuscular activation. While previous meta‐analyses have highlighted comparable hypertrophy and smaller strength gains with LL‐BFR compared to HL training [3], few trials have investigated voluntary activation using neurophysiological methods. Our findings support the hypothesis that HL training remains superior for enhancing neuromuscular activation and explosive performance, and that a brief HL phase following LL‐BFR does not fully compensate for attenuated neural adaptations. Still, the magnitude of effects was small for voluntary activation, as baseline values were already high in this age group. In an exercise physiology context, this underscores the irreplaceable role of mechanical loading for optimizing neural drive, while still highlighting LL‐BFR as a clinically relevant preparatory tool when HL is temporarily contraindicated. Together with earlier reports showing that BFR can be applied safely and effectively in rehabilitation and older populations, our results suggest a progressive model: initiating with LL‐BFR to build tolerance and strength, then transitioning to HL training to maximize neuromuscular adaptations.

## 5 Conclusion

This study compared the neuromuscular effects of LL‐BFR training and HL resistance training, including a subsequent HL phase applied in both groups. HL training consistently induced greater improvements in maximal strength and explosive performance, as well as small effects on voluntary activation than BFR training alone. Notably, a 2 week HL phase following 8 weeks of BFR training was insufficient to fully compensate for the attenuated neural adaptations seen in the BFR group.

These findings underscore that mechanical loading is a critical and irreplaceable stimulus for neuromuscular optimization. While BFR is a viable alternative when high loads are contraindicated, particularly for initial strength gains, its capacity to elicit sustained neural adaptations—especially in terms of voluntary drive—appears limited.

Future research should explore whether longer or progressively intensified HL phases following BFR can more effectively restore neuromuscular activation. Furthermore, trials involving populations such as older adults or clinical cohorts may help tailor sequential training strategies according to mechanical load tolerance and rehabilitation stage.

## Funding

This study was funded by the Freiwillige Akademische Gesellschaft (FAG) and the Research Fund Junior Researchers of the University of Basel. No external company, manufacturer, or outside organization provided technical or equipment support for this project.

## Ethics Statement

The study was approved by the Ethics Committee of Northwestern and Central Switzerland (EKNZ; Project‐ID: 2023‐01546) and conducted in accordance with the Declaration of Helsinki and Good Clinical Practice.

## Consent

All participants provided written informed consent prior to participation.

## Conflicts of Interest

The authors declare no conflicts of interest.

## Supporting information


**Table S1.** Descriptive statistics of all outcomes across time for each group. Number of valid measures (*N*); mean (SD = standard deviation).

## Data Availability

The anonymized dataset supporting the findings of this study is publicly available on the Open Science Framework at https://doi.org/10.17605/OSF.IO/FZ38R. Users are kindly asked to cite the dataset if used in any form.
