# Supplementary material for: Effects of Low‐Load Blood‐Flow Restriction Training Versus High‐Load Resistance Training on Neuromuscular Performance and Neuromuscular Activation
Source: Scand J Med Sci Sports. 2026 Jan 7;36(1):e70203. doi: 10.1111/sms.70203 (PMC12780320; doi:10.1111/sms.70203)
Supplement: Supplementary file 1 — Table S1. Descriptive statistics of all outcomes across time for each group. Number of valid measures (N); mean (SD = standard deviation). [file SMS-36-e70203-s001.docx]

Supplemental Table 1: Descriptive statistics with percentage change of all outcomes across time for each group only for complete cases (average change in % and standard deviation (SD)).

|  |  | **BFR** | | **HL** | |
| --- | --- | --- | --- | --- | --- |
|  | **Outcome** | **%change T0-T1 (SD)** | **%change T1-T2 (SD)** | **%change T0-T1 (SD)** | **%change T1-T2 (SD)** |
| Maximal isometric strength | Isometric leg extension [Nm] | -1.6 (10.4) | 3.8 (13.2) | 13.9 (24.0) | -1.4 (8.2) |
|  | Isometric leg extension [Nm/kg] | -0.9 (12.1) | 3.9 (13.0) | 11.8 (23.0) | -0.9 (8.5) |
|  | Isometric leg press [kN] | 5.9 (21.0) | 2.8 (18.2) | 8.7 (21.8) | 5.5 (28.7) |
|  | Isometric leg press [N/kg] | 2.5 (19.2) | 1.9 (19.6) | 4.9 (19.3) | 0.9 (23.1) |
| Maximal dynamic strength | Leg press 1-RM [kg] | 18.3 (1.8) | 3.0 (14.7) | 15.8 (1.8) | 23.9 (14.7) |
|  | Leg extension 1-RM [kg] | 20.5 (7.6) | 4.9 (9.5) | 31.3 (7.6) | 18.4 (9.5) |
|  | Leg flexion 1-RM [kg] | 16.2 (9.8) | 2.1 (10.2) | 30.1 (9.8) | 16.5 (10.2) |
| Maximal explosive strength | Rate of force development [Nm·150ms^-1^] | -15.0 (24.9) | 22.0 (85.2) | 17.4 (46.7) | -10.4 (39.2) |
|  | Countermovement-jump power [W/kg] | 1.4 (5.3) | 3.0 (3.8) | -2.2 (6.2) | 0.9 (5.1) |
|  | Countermovement-jump height [cm] | 5.1 (15.3) | 4.6 (10.8) | -1.6 (8.1) | 3.9 (10.6) |
| VA | Voluntary activation [%] | -1.1 (6.0) | 1.8 (10.7) | 2.9 (6.9) | 0.4 (6.8) |
